# UniProtKB amid the turmoil of plant proteomics research

**DOI:** 10.3389/fpls.2012.00270

**Published:** 2012-12-06

**Authors:** Michel Schneider, the UniProt Consortium, Sylvain Poux

**Affiliations:** ^1^Swiss-Prot, SIB Swiss Institute of Bioinformatics, Centre Médical UniversitaireGeneva, Switzerland; ^2^European Bioinformatics InstituteHinxton, UK; ^3^Protein Information Resource, Georgetown University Medical CenterWashington, DC, USA

**Keywords:** knowledgebase, protein, genome, complete proteome, proteomics

## Abstract

The UniProt KnowledgeBase (UniProtKB) provides a single, centralized, authoritative resource for protein sequences and functional information. The majority of its records is based on automatic translation of coding sequences (CDS) provided by submitters at the time of initial deposition to the nucleotide sequence databases (INSDC). This article will give a general overview of the current situation, with some specific illustrations extracted from our annotation of Arabidopsis and rice proteomes. More and more frequently, only the raw sequence of a complete genome is deposited to the nucleotide sequence databases and the gene model predictions and annotations are kept in separate, specialized model organism databases (MODs). In order to be able to provide the complete proteome of model organisms, UniProtKB had to implement pipelines for import of protein sequences from Ensembl and EnsemblGenomes. A single genome can be the target of several unrelated sequencing projects and the final assembly and gene model predictions may diverge quite significantly. In addition, several cultivars of the same species are often sequenced – 1001 Arabidopsis cultivars are currently under way – and the resulting proteomes are far from being identical. Therefore, one challenge for UniProtKB is to store and organize these data in a convenient way and to clearly defined reference proteomes that should be made available to users. Manual annotation is one of the landmarks of the Swiss-Prot section of UniProtKB. Besides adding functional annotation, curators are checking, and often correcting, gene model predictions. For plants, this task is limited to *Arabidopsis thaliana* and *Oryza sativa* subsp. *japonica*. Proteomics data providing experimental evidences confirming the existence of proteins or identifying sequence features such as post-translational modifications are also imported into UniProtKB records and the knowledgebase is cross-referenced to numerous proteomics resource.

## INTRODUCTION

The words “proteome” and “proteomic” were first coined by Marc Wilkins in 1996 (Wilkins et al., 1996a,b), with the former term defined as “the protein complement expressed by a genome” and the latter referring to its study. Since the first article describing the analysis of 27 proteins in *Escherichia coli*, several thousand publications have appeared describing proteomic studies of plants, and the development of appropriate databases and tools for the management and querying of this data are essential to maximize its utility.

As early as 1965, Margaret Dayhoff started collecting protein sequences in her “Atlas of protein sequence and structure” (Dayhoff et al., 1965), the first edition of which included a mere 65 proteins, which may seem impossibly small for bioinformaticians learning their trade in this era of “big data.” In 1984, the first computer protein sequence database was created by the Protein Information Resource (PIR) under the name “PIR-International Protein Sequence Database (PIR-PSD)”, while in 1986, an extended version based on the format of the European Molecular Biology Laboratory (EMBL) nucleotide sequence database was first freely distributed by Amos Bairoch under the name of Swiss-Prot. The first release of Swiss-Prot contained roughly 3,900 manually annotated proteins, growing gradually to a size of 83,000 proteins some 10 years later. By this time, the burgeoning growth in genome sequencing and high-throughput cDNA sequencing projects had already resulted in a situation where most newly identified proteins were not readily available in the database. To solve with this problem, TrEMBL, a computer-annotated supplement to Swiss-Prot, was launched. TrEMBL is composed of entries derived from the hypothetical translation of coding sequences (CDS) proposed by authors of sequence submissions to the International Nucleotide Sequence Data Consortium (INSDC) database. When the entries based on these CDS are curated they are subsequently included in Swiss-Prot, and are no longer available in TrEMBL. Since 1996, Swiss-Prot/TrEMBL is produced jointly by the SIB Swiss Institute of Bioinformatics (hereafter referred to as SIB) and the European Bioinformatics Institute (EBI).

Swiss-Prot/TrEMBL and PIR-PSD (Wu et al., 2003) continued to coexist independently until 2002, when the SIB, the EBI, and the PIR group at the Georgetown University Medical Center and National Biomedical Research Foundation joined forces to form the Universal Protein Resource (UniProt) Consortium^1^ (Apweiler et al., 2004). The goal of UniProt is to provide a single, centralized, authoritative resource for protein sequences and functional information, the cornerstone of which is formed by the combination of Swiss-Prot and TrEMBL, which was subsequently christened the UniProt KnowledgeBase, or UniProtKB.

The aim of this short review is not to give the current status of the annotation in UniProtKB, an information that can be found in the statistics provided with each release^2,3^, but to highlight some of the limitations and challenges encountered when producing a protein database and to describe some of the new features implemented to solve those problems. However in order to have an idea of the richness of the knowledgebase, it should be noted that, at the time of writing (UniProt release 2012_09), UniProtKB included 538,010 manually reviewed UniProtKB/Swiss-Prot entries and 26,079,526 computer-annotated UniProtKB/TrEMBL entries.

**FIGURE 1:**
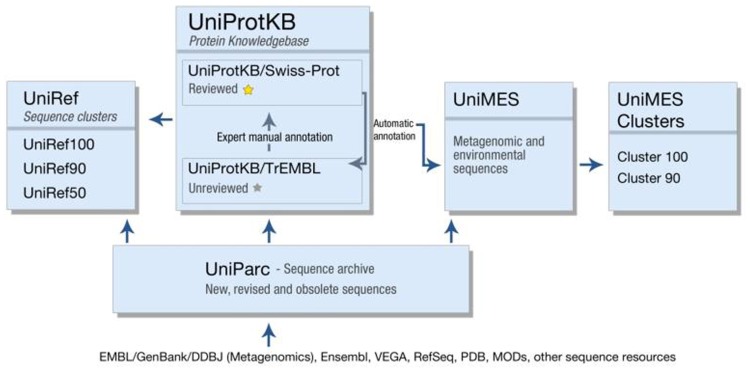
**The various databases produced by the UniProt consortium**.

In addition to UniProtKB, UniProt produces a number of other resources, each optimized for a different use (**Figure 1**). UniParc is an archive containing all publicly available protein sequences, including obsolete sequences from UniProtKB and other resources. UniRef uses the CD-HIT algorithm (Suzek et al., 2007) to cluster sequences from UniProtKB (including splice variants) and UniParc at 100, 90, or 50% identity, and selects a representative sequence from each cluster. UniRef clusters are intended for comprehensive and fast sequence similarity searches, providing high coverage of the available sequence space while reducing redundancy. Finally, UniMes is a distinct repository of metagenomic and environmental sequences, the precise taxonomic origin of which is unknown.

The representation of sequence variants within UniProtKB is complex, and deserves a special mention here. When sequence submitters provide annotated CDS as part of an INSDC submission, each of those distinct protein sequences is instantiated within UniProtKB/TrEMBL as a separate record. This applies equally to protein isoform sequences produced from a single gene, which will be initially present in distinct UniProtKB/TrEMBL records, one record per isoform. When individual isoform sequences from UniProtKB/TrEMBL records are subsequently reviewed by UniProt curators, they are merged into a single UniProtKB/Swiss-Prot record. Hence, a single UniProtKB/Swiss-Prot record may contain the putative translation(s) of all distinct isoforms that were available at the time of curation. Note that in addition to the merging of isoforms, the curation of UniProtKB/Swiss-Prot may also require the separation of distinct genes into distinct records. This is because identical protein sequences within a given taxon are merged during the production of UniProtKB/TrEMBL, meaning that a given UniProtKB/TrEMBL record may actually include (the identical products of) more than one gene. When such records are curated in UniProtKB/Swiss-Prot they are generally “demerged,” with the products of each distinct gene assigned to one individual record. This means that the exact same protein sequence may occur more than once for any complete proteome in UniProtKB/Swiss-Prot.

This demerging procedure is relatively new, and in the past identical protein sequences from distinct genes were also placed in a single UniProtKB/Swiss-Prot record – these are now being actively demerged. To give one example, UniProtKB/Swiss-Prot records P0DI10 (PER1_ARATH, At1g05240) and Q67Z07 (PER2 ARATH, At1g05250) describe identical peroxidase sequences of *Arabidopsis thaliana*, this family being extremely widespread in *A. thaliana*, with more than 70 members. These identical protein sequences are now present in distinct UniProtKB/Swiss-Prot records, as they derive from different genes – “one gene, one entry.” If we examine the history of one of these records, P0DI10^4^, we can see that this is a newly-created record that replaced UniProtKB record Q96506. Analysis of the history of this record in turn^5^ shows that this record originally represented both genes^6^, and was subsequently demerged. A number of notable exceptions to this general rule of “one gene-one record” can be found in UniProtKB/Swiss-Prot, such as the histones, where demerging has not been performed. The reasons for these exceptions are largely pragmatic: assigning functional annotation to individual histone genes, and maintaining those annotations in a consistent state over hundreds of identical UniProtKB records, is an extremely challenging task.

## COMPLETE PROTEOMES

Most proteomic analyses include an identification step which involves searching a protein sequence database for potential matches to the identified peptides (Nesvizhskii and Aebersold, 2005). Groups of matching proteins are then analyzed and inferences are drawn about the possible composition of the proteins in the original sample. The correctness of such inferences depends not only on the way in which peptide to protein sequence matches are interpreted, a subject which is outside the scope of this review, but also on the degree of completeness and accuracy of the protein sequences in the protein sequence database.

To support proteomics applications (and other studies of whole cellular systems), UniProtKB provides complete proteome sets. Each complete proteome includes the entire set of proteins that could potentially be expressed by the complete genome sequence of an individual organism. UniProtKB complete proteomes may include both manually reviewed (UniProtKB/Swiss-Prot) and unreviewed (UniProtKB/TrEMBL) records that describe protein sequences with variable levels of experimental support, ranging from those protein sequences that have been confirmed to exist through prior proteomic experiments to those whose existence is entirely hypothetical. An indication of the available evidence for the existence of each protein is given by a “Protein Existence” (PE) line that can take a value between 1 (evidence at protein level) and 5 (Uncertain or possible pseudo-gene product). Criteria used to assign a PE level to entries are described in a document file available on the UniProt web site^7^. Each UniProtKB record from a complete proteome is tagged with a specific keyword “Complete proteome,” which can be used in combination with specific taxonomic identifiers to query UniProtKB for complete proteome sets. The number of complete proteomes is increasing at each UniProtKB release and an up-to-date list can be found at: http://www.uniprot.org/taxonomy/complete-proteomes. The methods for the retrieval of complete proteomes is detailed in http://www.uniprot.org/faq/15.

As mentioned in the introduction, UniProtKB/TrEMBL includes entries derived from the hypothetical translation of CDS proposed by authors in sequence submissions to the INSDC database. This applies to whole genome submissions too, meaning that many complete proteomes in UniProtKB will be associated with a corresponding whole genome submission. For those genome sequences for which the CDS are not available through INSDC but stored in specialized databases, such as *Sorghum bicolor* or *Brachypodium distachyon* for example, protein sequences are imported from the EnsemblGenomes database (Kersey et al., 2012), which disseminates annotated genomes for a number of model organisms and model organism databases (MODs) in the Ensembl framework (Youens-Clark et al., 2011).

In the case of *A. thaliana*, the most recent re-annotation of the latest assembly of the genome by The Arabidopsis Information Resource (“TAIR10”) was imported from their web site^8^. During this process, curators from UniProt and TAIR collaborated extensively to resolve discrepancies and more than 98.5% of the protein sequences are now identical in both sets.

The criteria used to include a proteome in the “complete proteome” set are multiple. First of all, the complete genome should be sequenced, assembled, and publicly available. That already explains why some important crops such as wheat are not yet in the complete proteome set. Then, the genome assembly should be stable and the gene models reasonably predicted. The maize proteome failed at this step: the original 110,028 members of the “working gene set” annotated on the assembly version “RefGen_v2” have been now filtered to 63,540 gene models organized in the 39,656 members of the “filtered gene set.”^9^ Fortunately, a new assembly “B73 RefGen_v3” was built and is currently being annotated. Once this new data is available from EnsemblPlant, it will be reconsidered for inclusion in the complete proteome set.

## REFERENCE PROTEOMES

**FIGURE 2:**
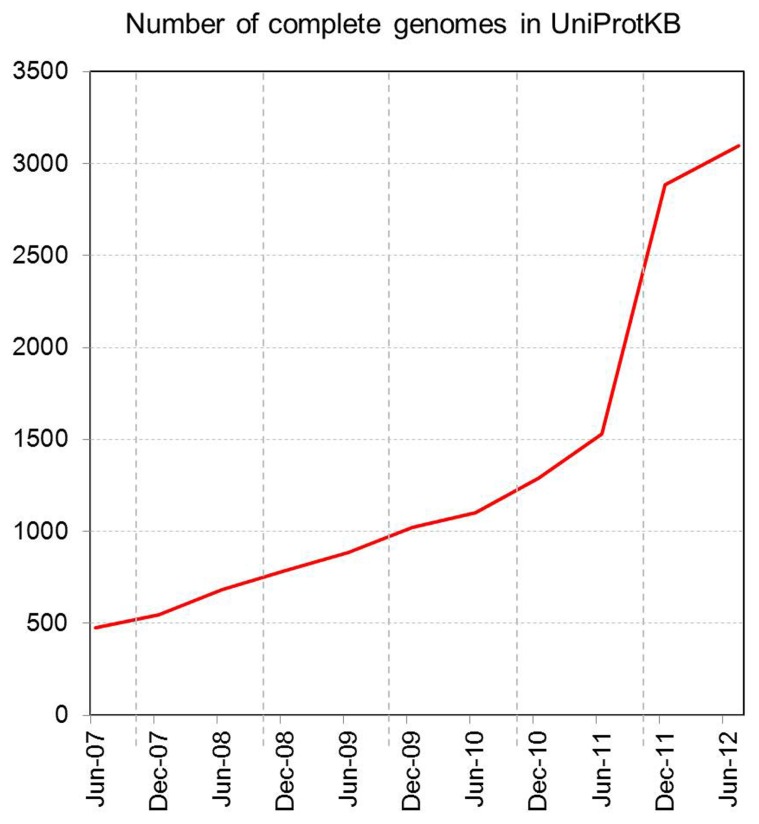
**Number of complete genomes in UniProtKB**.

**FIGURE 3:**
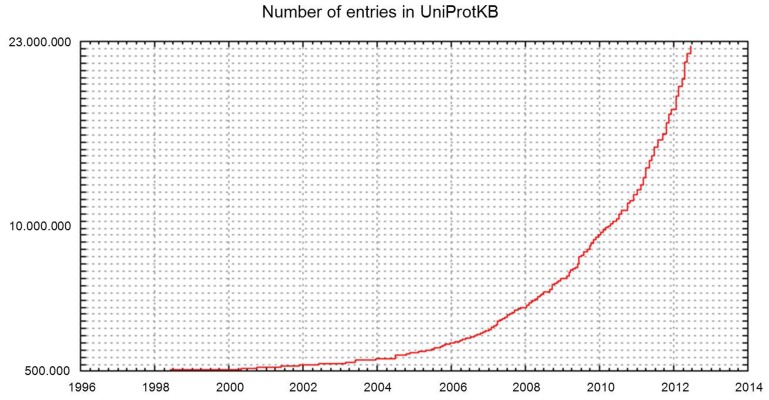
**Number of entries in UniProtKB**.

Increasing access to high-throughput sequencing technologies and their continuing development have led to unparalleled rates of growth in the number of available complete sequenced genomes, and of databases such as UniProtKB that store them (**Figures 2 and 3**).

Large-scale genome sequencing surveys are being performed over a diverse range of taxonomic scopes: projects like Microbial Earth^10^ aim to sample the entire taxonomic diversity of entire kingdoms, while the 1001 Genomes Project^11^ aims to discover the whole-genome sequence variation in 1001 cultivars (or accessions) of a single species, namely *A. thaliana* (Weigel and Mott, 2009; Ledford, 2011).

Irrespective of the source and scope of the data that is subsequently submitted to UniProtKB/TrEMBL, it is critically important to organize this data in a way that allows users to effectively navigate this information in useful ways. One approach adopted by UniProt to meet this challenge is to define a set of “reference proteomes” which are “landmarks” in proteome space^12^. The records that constitute these proteomes are tagged with the keyword “Reference proteome,” and the corresponding proteomes retrieved by searching for taxa bearing this keyword in a similar way to “Complete proteomes.”^13^ Reference proteomes provide a representative cross-section of the taxonomic diversity to be found within UniProtKB, including proteomes of well-studied model organisms and other proteomes of interest for biomedical and biotechnological research. In most cases only a single reference proteome is defined for each species, meaning that the genome and proteome sequences of the 1001 cultivars of *A. thaliana* would (if submitted as complete genome assemblies to INSDC) be classified separately: one cultivar – most probably cv. Columbia – would be defined as a “Reference proteome” while the remaining 1000 cultivars would be classified as “Complete proteomes.” In compliance with the current UniProtKB/TrEMBL production rules, every protein sequences originating for the same plant species are be grouped under a single taxonomic identifier (NCBI_TaxID = 3702 for *A. thaliana* for example), irrespective of the cultivar of origin. To allow the retrieval of the individual proteomes, UniProt is planning to introduce a method based on the use of unique proteome identifiers. The list of the UniProt reference proteomes can be found at: http://www.uniprot.org/taxonomy/complete-proteomes.

## VARIABILITY AND INSTABILITY OF THE DATA EXTRACTED FROM WHOLE GENOME SEQUENCING PROJECTS

One issue knowledgebases have to deal with is the instability of the genome assemblies and of the gene model predictions. As an illustration, in 2005 The Institute for Genomic Research (TIGR) predicted a content of 43,719 genes in *Oryza sativa* subsp. *japonica* cv Nipponbare (Yuan et al., 2005) while 7 years later, the estimate is 39,045 loci resulting in 49,066 different gene models^14^. Moreover, a same genome can be the target of several sequencing projects that will result in different final assemblies and different number of predicted genes. *Oryza sativa* subsp. *japonica* cv Nipponbare for example, was subjected to a whole-genome shotgun (WGS) sequencing by Syngenta that led to the prediction of 45,824 genes while the International Rice Genome Sequencing Project (IRGSP) using a clone-by-clone sequencing approach predicted 43,635 genes. Of these two sets of predictions only 41,225 were common, i.e., sharing at least 50% of their coding regions (Yu et al., 2005).

By the end of last year, and as a first step toward unification, the MSU Rice Genome Annotation Project, which took over the TIGR data, and the Rice Annotation Project Database (RAP-DB)/International Rice Genome Sequencing Project^15^ released a common final assembly of the rice genome. However, the gene model predictions still differ, with a prediction of a total of 49,066 and 50,444 coding gene models respectively. Due to the way UniProtKB/TrEMBL is produced, and in order to be as comprehensive as possible, both sets of proteins are currently merged into a single complete proteome composed of 63,553 records.

In the same direction, the genome sequence of *Oryza sativa* subsp. *indica *cv 93-11 is very similar to the one from *Oryza sativa *subsp. *japonica* cv Nipponbare, but the gene models proposed by the Beijing Genome Institute (BGI)^16^ who did the sequencing differ extensively, both in number (40,745) and in structure.

As already mentioned, one general trend is to deposit the raw sequence of a complete genome to the nucleotide sequence databases (INSDC) while the gene model predictions and annotations are kept in a separate, specialized MOD. This way of doing has one main drawback: the durability of such MODs is not granted and a common challenge for those resources is finding financial support for maintenance and development (Chandras et al., 2009). Even well established and praised databases such as TAIR are not immune from financial turmoil, leading, in this particular case, to an irredeemable closure of the resource in 2013 (Abbott, 2009). The complete list of related publications and the GO annotation provided by TAIR will continue to be displayed in the corresponding UniProtKB entries. A new Arabidopsis Information Portal (AIP) that will include all the functionalities currently found at TAIR is under construction by the The International Arabidopsis Informatics Consortium (2012).

## UNIPROTKB ANNOTATION: SEQUENCE CURATION

Gene-build process usually performed on newly sequenced genomes combines homology-based and *ab initio* methods, but it nevertheless results quite frequently in erroneous gene model predictions. As a consequence, one important task provided by the UniProt curators is to check and improve the models proposed by the submitters by aligning them to published cDNA sequences or by comparing the proposed protein sequence to known orthologous or paralogous proteins. Since those checks and corrections are highly time consuming, UniProt plant curators limit this work to two model organisms, the monocot *Oryza sativa* subsp. *japonica* and the dicot *Arabidopsis thaliana*.

Beside gene models correction, UniProt curators are heavily involved in the manual annotation of small-scale papers describing only a limited number of proteins, but giving confirmation of the existence of specific gene products, including splicing isoforms. In addition to functional annotation, a special focus is put on the identification and the annotation of post-transcriptional modifications (PTMs) that are modifying the size and/or the mass of the various peptides composing the protein. These include transit peptides, processing sites, phosphorylation or glycosylation sites, modified amino acids, etc. A list of all the PTMs annotated in UniProtKB can be found at: http://www.uniprot.org/docs/ptmlist.

Variants observed between different plant cultivars are generally not incorporated into UniProtKB, but left in specialized databases such as the 1001 genomes project portal (Joshi et al., 2012) for *A. thaliana* variants. End users should easily make the correlation between the functional annotation attached to the reference proteome found in UniProtKB and the variant sequences stored at the 1001 genomes web site.

As the number of plant genomes completely sequenced is exploding, it becomes impossible to check and process all the data manually. In order to provide guidance to the users to identify the best suited set of data for their studies, UniProtKB is currently testing an annotation score for each entry and for each proteome. This measure of the intrinsic information content associated with a given entry or proteome will be put in production in the near future.

## PROTEOMICS RESOURCES AND REPOSITORIES

Availability of proteomics data and related meta-data is important to support published results and conclusions. Some journals already require, though with variable levels of stringency that raw data is uploaded in a public data repository such as Tranche (Smith et al., 2011). In a similar way, identified peptides can be submitted to dedicated databases like PRIDE (Csordas et al., 2012). A framework is under development by the ProteomeXchange consortium^17^ that will allow a dataset to be submitted to a central repository, where once associated with appropriate meta-data it gets a DOI (and therefore can be considered as citable information). This will permit external resources such as proteomics repositories and software developers to use and reprocess the data. It will also eventually allow UniProtKB to select data that is suitable for annotation purposes.

The need for improved quality control and standardization is widely recognized among the proteomics community (Eisenacher et al., 2011) and guidelines about the “minimum information about a proteomics experiment” (MIAPE; Taylor et al., 2007) have been established in addition to an increased stringency in journal submission guidelines.

Besides those repositories, numerous very valuable resources, each focused on a specific aspect like tandem mass spectra evidences, quantitative information, localization of phosphorylation sites, are available for plant proteomics such as ProMEX (Wienkoop et al., 2012), PhosPhAt, a plant phosphorylation site database (Arsova and Schulze, 2012), PaxDb (Wang et al., 2012), a meta-resource integrating information on absolute protein abundance levels across different organisms, including *A. thaliana*, MASCP Gator (Joshi et al., 2011), an aggregation portal for the visualization of *Arabidopsis* proteomics data or PPDB, the Plant Proteomics Database (Sun et al., 2009) to cite only a few. UniProtKB is cross-linked to several of those proteomics resources, including PRIDE, IntAct, ProMEX, PeptideAtlas, and PhosphoSite. A complete list of the cross-references, with bibliographic references, is available at: http://www.uniprot.org/docs/dbxref.

## UNIPROTKB ANNOTATION: PROTEOMICS DATA CURATION

The field of proteomics is also providing large amount of data that has to be dealt with. Publications and dataset reports from large-scale proteomics experiments constitute a rich set of experimental evidences confirming the existence of proteins as well as identifying sequence features such as post translational modifications. However, they exhibit highly variable formats, and different levels of reliability and confidence. This is due to the heterogeneous nature of proteomics experimental protocols on one hand, and to the use of different methods for the analysis and interpretation of results on the other hand. Many high-throughput proteomics data sets are reported using a 1% false-positive identification rate. Incorporating these data in their entirety in a database such as UniProtKB has a cumulative effect, through which the overall proportion of false identification will increases in the knowledge base with the number of incorporated datasets (Olsen and Mann, 2011). Potentially this might negatively impact further research areas (White, 2011).

To address this issue, UniProtKB is implementing a stringent procedure for selecting the data to be incorporated in the database, increasing by this way the quality and the reliability of the data imported from large-scale proteomics experiments. This important subject deserves a complete and separate article by its own, which will be submitted in a near future.

## CONCLUSION

While the increasing flow of incoming data becomes a flood, and even recently a tsunami, databases should evolve and adapt themselves to this new environment in order to be able to provide the right tools required for coherent use of proteomics in plant biotechnology research. If it is well recognized that good annotation in plant proteomics is a prerequisite for good data interpretation and analyses, it is challenging to produce and maintain a high quality protein database.

Strong efforts should be made to implement quality control and standardization procedures at the level of the data production already and international bodies such as the Human Proteome Organization (HUPO) or the International Plant Proteomics Organization (INPPO; Agrawal et al., 2011, 2012) have an important role to play in advocating and promoting their enforcement in research labs. Improving the quality and reliability of the original data would help UniProtKB capturing and integrating proteomic-based information in its records, allowing us to maintain a high quality knowledgebase.

Since most proteomic analyses rely on an identification step based on searching a protein sequence database for potential matches to the identified peptides, the accuracy of the results is strongly correlated with the selection of the reference database to be used. The effect of changing the database used can be dramatic (Knudsen and Chalkley, 2011). UniProtKB, and specially its manually annotated section, Swiss-Prot, strives to provide the best possible clean and “safe” data to be used for the identification of proteins. That includes both a broad coverage of genome or taxon space and a high number of manually checked gene models for the two plant models, *A. thaliana* and *O. sativa *subsp. *japonica*. We have here a clear win-win situation: UniProtKB can give a comprehensive set of proteins that should be used for accurate peptides identification while the resulting proteomics data will be used to continually complete and improve the content of the protein knowledgebase.

## Conflict of Interest Statement

The authors declare that the research was conducted in the absence of any commercial or financial relationships that could be construed as a potential conflict of interest.

## Acknowledgments

Many thanks to all the UniProtKB/Swiss-Prot team, especially to Lydie Bougueleret, Pierre-Alain Binz, and Alan Bridge for critical reading of this manuscript and helpful suggestions.

UniProt is mainly supported by the National Institutes of Health (NIH) grant 1 U41 HG006104. Additional support for the EBI’s involvement in UniProt comes from the NIH grant 2P41 HG02273. Swiss-Prot activities at the SIB are supported by the Swiss Federal Government through the Federal Office of Education and Science and the European Commission contracts SLING (226073), Gen2Phen (200754), and MICROME (222886). PIR’s UniProt activities are also supported by the NIH grants 5R01GM080646-07, 3R01GM080646-07S1, 5G08LM010720-03, and 8P20GM103446-12, and the National Science Foundation (NSF) grant DBI-1062520.

## Footnotes

1 http://www.uniprot.org

2 http://web.expasy.org/docs/relnotes/relstat.html

3 http://www.ebi.ac.uk/uniprot/TrEMBLstats/

4 http://www.uniprot.org/uniprot/P0DI10?version=*

5 http://www.uniprot.org/uniprot/Q96506?version=*

6 http://www.uniprot.org/uniprot/Q96506.txt?version=102

7 http://www.uniprot.org/docs/pe_criteria

8 http://www.arabidopsis.org/

9 http://ftp.maizesequence.org/current/README.txt

10 http://genome.jgi.doe.gov/programs/bacteria-archaea/MEP/index.jsf

11 http://www.1001genomes.org/

12 http://www.uniprot.org/faq/47

13 http://www.uniprot.org/faq/15

14 http://rice.plantbiology.msu.edu/

15 http://rapdb.dna.affrc.go.jp/

16 http://rise2.genomics.org.cn/page/rice/index.jsp

17 http://www.proteomexchange.org
